# Understanding etiology of chromosome 21 nondisjunction from gene × environment models

**DOI:** 10.1038/s41598-021-01672-x

**Published:** 2021-11-17

**Authors:** Pinku Halder, Upamanyu Pal, Agnish Ganguly, Papiya Ghosh, Anirban Ray, Sumantra Sarkar, Sujay Ghosh

**Affiliations:** 1 Cytogenetics and Genomics Research Unit, Department of Zoology, University of Calcutta, Kolkata, West Bengal India; 2 Department of Zoology, Bijoy Krishna Girls’ College (Affiliated to University of Calcutta), Howrah, West Bengal India; 3 Department of Zoology, Bangabasi Morning College (Affiliated to University of Calcutta), Kolkata, West Bengal India; 4 Department of Paediatric Medicine, Diamond Harbour Government Medical College and Hospital, Diamond Harbour, West Bengal India

**Keywords:** Genetics, Risk factors

## Abstract

Maternal risk factors and their interactions with each other that associate chromosome 21 nondisjunction are intriguing and need incisive study to be resolved. We determined recombination profile of nondisjoined chromosome 21 and maternal genotypes for four selected polymorphic variants from the folate regulators genes stratifying the women according to the origin of segregation error and age at conception. We conducted association study for genotype and maternal addiction to smokeless chewing tobacco, usually chopped tobacco leaves or paste of tobacco leaves with the incidence of Down syndrome birth. Additionally, we designed various logistic regression models to explore the effects of maternal genotype, maternal habit of smokeless chewing tobacco, maternal age at conception and all possible interactions among them on chromosome 21 nondisjunction. We found folate regulator gene mutations are associated with maternal meiosis II error. Regression models revealed smokeless chewing tobacco and folate polymorphic/mutant risk genotype interact with each other to increase the risk of reduced and single peri-centromeric recombination events on chromosome 21 that nondisjoined at meiosis II in the oocytes and the effect is maternal age independent. We inferred maternal folate polymorphic/mutant risk genotypes and habit of smokeless chewing tobacco interact with each other and increase the risk of meiosis II error in oocytes in maternal age-independent manner.

## Introduction

Down Syndrome (DS) or Trisomy 21 is the most frequent genetic form of intellectual disability in human and is caused by the failure of chromosomes to separate properly during meiosis, the error known as chromosome nondisjunction (NDJ). Among overwhelming majority of cases NDJ occurs at meiosis I^1^ of oogenesis^2^.

Maternal risk for DS child birth is multifactorial and includes factors of both genetic and environmental origin^1,3^ and challenges faithful chromosome segregation either maternal age-dependent manner or a stochastic age-unrelated fashion^4,5^. Advanced maternal age^6^ and altered recombination profile of chromosome 21 (Ch21) are two risk factors that have been identified to be associated with DS birth risk in previous studies^7^. Reduction in recombination frequency of pairing Ch21 homologues and altered chiasma positions are two well-recognized ‘molecular’ correlates of improper chromosome segregation^8^. In addition, some prospective candidates for epidemiological risk factors have been reported to be associated with DS birth^9–11^, though results are contradictory. Among them, maternal periconceptional drinking and smoking are common habits of women in western countries. Among Indian women use of Smokeless Chewing Tobacco (SCT) is highly prevalent. Smokeless chewing tobacco includes chopped tobacco leaves and paste of tobacco leaves popular in South Asian Countries. Studies conducted on human and mice reported genotoxic effects of smoking and use of tobacco on reproductive health and fertility^9,10,12^. Previously, we analysed the association of SCT as the epidemiological risk factor of DS birth^13^ and found SCT was a significant risk factor for reduction in recombination frequency on Ch21q in maternal age-dependent manner. When tested for spatial distribution of single recombinant events on 21q, we did not find any difference between the SCT users from the SCT non-users. Nevertheless, it is still obscure whether SCT interacts with any other risk factors when co-occur together or impose any synergistic effects in association with any other maternal genetic factors to imperil ideal chromosome segregation process. This issue could only be resolved by analysing ‘gene × environment (G × E) models’ considering maternal genotype as genetic risk component on one hand and SCT use as environmental or epidemiological risk factor on the other hand. The G × E model is increasingly being explored in many studies on intellectual disabilities^14,15^. A substantial amount of human and animal data has proved that environmental or epidemiological influence can exacerbate the symptoms of many neurological disorders having susceptible genetic background^16,17^. Keeping this fact in mind we were curious to look into whether maternal exposure to SCT interacts with maternal genetic risk factors like polymorphic variants of any gene that has been reported previously as risk for Ch21 NDJ to cause chromosome segregation error. Several polymorphic variants of many maternal genes exhibited specific association with either MI or MII segregation pattern as revealed from recent GWAS on the population from USA^18^.

Polymorphisms of maternal folate metabolic regulators have been identified as risk factors associated with DS birth^19^. It is hypothesized that deficiency in folate pathway may perturb the centromeric methylation pattern which is needed for its attachment with spindle and chromatid separation^20^. Methylenetetrahydrofolate reductase (MTHFR), Methionine Synthase Reductase (MTRR), Methionine Synthase (MTR) genes are key regulators of folate metabolism pathways and polymorphic variants of these genes have been reported as maternal risk factors for DS birth in many ethnic populations, though results are contradictory^21–23^. Maternal MTHFR C677T polymorphism was detected to be associated with increased risk of DS in the USA, Canadian and Brazilian populations^21,22,24,25^. Further, an association between the MTRR A66G genotype with the MTHFR C677T variant^21,25,26^ as risk factor of DS child birth was evident while such association remains unidentified in Sicilian population^23^. On other hand, Sicilian study reported for first time the effect of MTR A2756G variant on the risk of DS and the effect was reported to be stronger when associated with MTRR A66G polymorphism. MTHFR A1298C variant was associated with increased risk of DS in Portuguese population^21^. In our previous study^27^ we have reported association between maternal folate metabolic regulator polymorphisms and increase risk of MII NDJ of Ch21 in oocyte among the Bengali speaking women from West Bengal, India. In the present study, we have designed ‘G × E models’ to test how the genetic and the environmental or epidemiological factors interact to predispose women to have meiotic NDJ error and subsequent DS birth considering maternal habit of SCT use as environmental challenge and maternal genotypes of selected folate regulator variants as genetic risk factors. This study is designed following our previous analyses^13^ in which we tested various regression models considering maternal age at conception and SCT use as predictors and meiotic stage of NDJ error and recombination pattern as outcome. In addition to those previously analysed predictors, we have now included maternal genotypes of folate metabolic regulator genes as third predictor. This study is important as it will reveal the complicated multidimensional interactions among the various risk factors that synergistically create a predisposing ambience of Ch21 NDJ in maternal ovary.

## Result

### Sample population stratification

A total of 1294 families each with unambiguous maternal origin of supernumerary Ch21 were included as case families. Among them 956 and 338 families exhibited MI and MII origin of the extra Ch21, respectively. 870 families with healthy child were included as control families. Within MI error group 283 and 673 families were SCT user and non-user. Among MII error group 180 and 158 families were SCT user and non-user.

### Epidemiological and clinical attributes of the study participants

We performed t-tests to evaluate the differences between mean maternal and age at conception, preconception maternal folic acid intake amount and chi square (χ2) test was used to compare other epidemiological parameters between MI and MII, case and control groups. The estimated mean maternal age at conception in the MI and MII error groups having DS child were 30.15 ± 4.1 years (mean ± SD) & 30.02 ± 3.4 years, as compared with control group 29.91 ± 3.9 years (*P* = 0.20 for MI vs Control, *P* = 0.65 for MII vs Control) (Table 1). The mean age of control women with folate regulator wild-type genotypes (folate-WT) was 33.09 ± 3.1 years which is similar to the estimate 32.90 ± 2.3 years for the MI women with DS child (*P* = 0.13) and 33.10 ± 3.3 years for the MII women with DS child (*P* = 0.96). The mean age of control women with folate polymorphic/mutant risk genotype (folate-MUT) was 31.91 ± 3.5 years, which is again concordant with the estimated mean age 32.01 ± 4.4 years for MI mothers (*P* = 0.59) and 31.60 ± 3.8 years for MII mothers (*P* = 0.18) having DS child. When we compared the mean age of the women in MI risk variant group with the MII risk variant group, the difference remained insignificant (*P* = 0.13). Moreover, the meiotic outcome groups did not exhibit significant difference in mean paternal age of conception when compared to control group (*P* = 0.24 MI vs control; *P* = 0.72 for MII vs control). As we dealt with maternal errors and maternal genotypes only, we did not analyze paternal genotypes. The other epidemiological parameters remain concordant between MI and MII case and control groups.

**Table 1:** Demographic and epidemiological detail of participating families in the study.

Criteria	DS bearing women		Control women
	MI	MII	
Families participated in the study	956	338	870
Mean maternal age at conception (all referred cases) [year ± SD]	30.15 ± 4.10	30.02 ± 3.40	29.91 ± 3.90
Mean maternal age of women with Folate regulator wild-type genotypes [year ± SD]	32.90 ± 2.30	33.10 ± 3.30	33.09 ± 3.10
Mean maternal age of women with folate polymorphic/mutant risk genotype [year ± SD]	32.01 ± 4.40	31.60 ± 3.80	31.91 ± 3.50
Mean paternal age at conception. (all referred cases) [year ± SD]	32.80 ± 4.10	32.90 ± 7.20	33.01 ± 3.40
Folic acid intake amount (mean ± SD μm/day)	432.5 ± 6.1	355.7 ± 7.2	536.5 ± 2.5
**Socio-economic condition of families**			
Low [Frequency]	295 [0.31]	186 [0.55]	323 [0.37]
Middle (INR 30,000–50,000/month) [Frequency]	517 [0.54]	118 [0.35]	443 [0.51]
High [Frequency]	144 [0.15]	34 [0.1]	104 [0.12]
**Locality**			
Kolkata metropolitan [Frequency]	698 [0.73]	213 [0.63]	609 [0.70]
Suburbs [Frequency]	172 [0.18]	71 [0.21]	174 [0.20]
Rural [Frequency]	86 [0.09]	54 [0.16]	87 [0.10]
**Religion**			
Hindu [Frequency]	851 [0.89]	247 [0.73]	792 [0.91]
Islam [Frequency]	86 [0.09]	71 [0.21]	52 [0.06]
Others [Frequency]	19 [0.02]	20 [0.06]	26 [0.03]
**Genotype status**			
Wild-type genotype [Frequency]	908 [0.95]	267 [0.79]	808 [0.93]
Folate polymorphic/mutant risk genotype [Frequency]	48 [0.05]	71 [0.21]	62 [0.07]

*DS* Down syndrome, *MI* Meiosis I, *MII* Meiosis II, *SD* standard deviation. t-tests were used to test for differences between mean maternal and age at conception, preconception maternal folic acid intake amount, chi square (χ^2^) tests were used to compare other epidemiological parameters between MI and MII case and control groups. *P* value < 0.05 was considered statistically significant.

### Frequencies of SCT use in control and case groups by age categories

A total of 1294 families with child having DS (Case) and 870 families with healthy child (Control) were included in this study. We categorized each of this group as ‘SCT non-user’ (case 0.64 vs control 0.83) and ‘SCT user’ (case 0.36 vs control 0.17) sub-categories according to the declared SCT use status in epidemiological record. We performed Fisher’s exact test for 2 × 2 contingency tables for comparing the frequencies of SCT use status in case and control groups and *P* value < 0.05 was considered as statistically significant. We found significant difference in maternal SCT use with odds in favour of the case mothers (OR = 2.772, 95% CI = 2.245—3.424, *P* < 0.0001) (Supplementary Table S1). We further stratified the case and the control women according to their age at conception as young age group (≤ 28 years), middle age group (29 to 34 years) and old age group (≥ 35 years) and tested if there was any significant difference in maternal SCT use pattern across the age. The frequency of SCT use between control and case group differs significantly in the young age group (OR = 1.996, 95% CI = 1.468- 2.634, *P* < 0.0001) and the middle age group (OR = 1.458, 95% CI = 1.056–2.015, *P* = 0.024) but not in the old age group (OR = 1.133, 95% CI = 0.760–1.686, *P* = 0.608) (Supplementary Table S2).

### Frequency of folate regulator polymorphism in case–control and meiotic outcome groups

We calculated frequency of all tested polymorphic variants of folate metabolic regulators and considered the mothers who bear any of the four tested polymorphisms together as a group and found ~ 5 folds increased odds in favour of case mothers over the controls (OR = 5.338; CI = 4.015–7.097; *P* < 0.0001). Out of the 1294 case mothers 956 and 338 were detected as MI and MII error categories, respectively. We have tested all four polymorphic variants, namely MTR A2756G, MTRR A66G, MTHFR C677T & MTHFR A1298C for all MI and MII case samples. The frequency of polymorphic variants in the meiotic outcome groups is given in Table 2. All four tested polymorphic variants exhibited strong association with maternal meiosis II NDJ error and this reconfirms the result that we obtained in our previous study^27^. However, frequencies of all four polymorphic variants were estimated negligible in the MI NDJ group and so we considered only the MII group as the ‘case sample’ for further analyses that include association study (Supplementary Table S3) and logistic regression modelling.

**Table 2:** Genotype frequency of folate regulator mutants MTR A2756G, MTRR A66G, MTHFR C677T and MTHFR A1298C among the DS bearing women stratified by meiotic errors.

Variants	Genotype	MI NDJ (N = 956)	MII NDJ (N = 338)	χ^2^	OR	95% CI, *P* value
MTR A2756G	AA	0.9	0.57	–	1	Reference
	AG	0.08	0.41	195.84	8.15	5.92–11.23, < 0.0001
	GG	0.02	0.02	0.737	1.64	0.68–3.96, 0.391
MTRR A66G	AA	0.93	0.53	–	1	Reference
	AG	0.07	0.44	254.87	11.05	7.94–15.36, < 0.0001
	GG	–	0.03	41.9	104.06	6.07–1785.2, < 0.0001
MTHFR C677T	CC	0.95	0.82	–	1	Reference
	CT	0.04	0.15	48.09	4.4	2.83–6.84, < 0.0001
	TT	0.01	0.03	6.29	3.28	1.35–7.96, 0.012
MTHFR A1298C	AA	0.94	0.35	–	1	Reference
	AC	0.06	0.55	448.31	24.86	17.46–35.40, < 0.0001
	CC	–	0.1	200.73	523.76	31.88–8605.1, < 0.0001

Chi square (χ^2^) test was performed to compare MI: MII ratios; Odds ratios with respective 95% Confidence Interval (CI) were calculated to find out the association of genotypes with meiotic outcome groups and two-tailed *P* value < 0.016 is considered statistically significant after Bonferroni correction test.The genotype data of MI group used as reference. *MI* Meiosis I, *MII* Meiosis II, *NDJ* nondisjunction, *N* number of individuals, *χ*^*2*^ chi square, *OR* odd ratio, *CI* confidence interval.

Some of the participating women carried more than one tested folate polymorphic genotypes and for them we calculated synergistic effect through gene–gene interactions models for all four tested variants. All the possible genotype combinations of any two given loci at a time were tested taking wild type genotype as reference (Supplementary Table S4).

Further, we have designed hypothetical models to evaluate the additive risk of maternal genotypes in combination with all the tested variants (Supplementary Table S5) and found gradual increase in risk of DS birth with increasing number of risk alleles in the tested loci. When all four loci carry risk alleles together either in homozygous or heterozygous state in the tested models, a ~ 17 folds odds in favour of MII errors was evident (*P* < 0.0001). When three out of four loci carried respective minor allele together, we estimated ~ 9 folds increased odds in favour of MII error (*P* < 0.0001).

### Interactions among risk factors

We stratified the participating case mothers into three age categories, based on maternal age at the time of conception following our previous definition^5^: young age group (≤ 28 years), middle age group (29–34 years) and old age group (≥ 35 years). For all the analyses, the maternal age of conception was considered as proxy for oocyte age as direct estimation of the later was beyond the scope of the present study. We used binary logistic regression and linear regression models to study a variety of questions regarding interaction among genetic risk factors i.e., amount of recombination, location of recombination, folate regulator gene mutations and epidemiological risk factor i.e., maternal SCT use and their association with maternal age at conception of DS foetus. Our analyses and statistical models were designed to address the following principal questions: (1) Does any significant difference exist in smokeless chewing tobacco (SCT) use among the cases and controls, and does this depend on maternal age and maternal folate regulator mutations or polymorphisms? (2) Considering only cases, is there any difference in SCT use among the MI and MII error groups, and does this depend on maternal and folate regulator mutations or polymorphisms? (3) Considering MI and MII cases separately, is there any relation between SCT use and the amount of meiotic recombination and does this depend on maternal age and does folate regulator mutation or polymorphisms have any effect on it? (4) Again, considering MI and MII cases separately, is there any relation between SCT use and the location of meiotic recombination and does this depend on maternal age and folate regulator polymorphisms? All these models explored the risk factors separately as well as considered them together to find any interaction among them that predispose women for having DS pregnancy.

### Model I: effects of SCT and genotype in cases versus controls

In case–control analyses we considered maternal age, maternal genotype and SCT use as variables and DS birth as outcome. The frequency of errors in case and control groups stratified by maternal folate-MUT, maternal age at conception and SCT use status is represented in the Table 3. We found maternal age, folate-MUT and SCT use as significant predictors (*P* < 0.01) with young age group having more frequent meiotic errors and the old age group having the least frequent errors (Table 3). In logistic regression analyses we tested various models (Table 3) of interactions among the predictors. We found significant elevated odds in favour of case women in the interaction models, namely young age group × SCT use × folate-MUT, middle age group × SCT use × folate-MUT and old age group × SCT use × folate-MUT. This suggests both the epidemiological and genetic risk factors together increase risk of NDJ error in all the age. We obtained intriguing result in the model old age group × SCT non-user × folate-MUT which increases odds nearly 6 folds (*P* < 0.01) in favor of case women.

**Table 3:** Distribution of case–control women stratified by folate regulator genotype, SCT use status and maternal age at conception show interactions among various risk factors and their association with case and control women.

	Genotype	SCT use	Age	Number (%)	Regression analysis			
					Interactions (maternal age × SCT use status × polymorphism status)	OR	95% CI	*P* value
Case (N = 1294)	Folate-MUT (N = 376)	User (N = 214)	Young age group (≤ 28 yrs.)	0.42	Young age group × SCT non-user × folate-WT	Reference		
			Middle age group (29–34 yrs.)	0.32				
			Old age group (≥ 35 yrs.)	0.26				
		Non-user (N = 162)	Young age group (≤ 28 yrs.)	0.42	Young age group × SCT non-user × folate-MUT	1.56	0.98–2.49	0.063
			Middle age group (29–34 yrs.)	0.31				
			Old age group (≥ 35 yrs.)	0.27	Young age group × SCT user × folate-WT	1.22	0.86–1.74	0.267
	Folate-WT (N = 918)	User (N = 250)	Young age group (≤ 28 yrs.)	0.46				
			Middle age group (29–34 yrs.)	0.33	Young age group × SCT user × folate-MUT	7.59	3.61–15.98	< 0.01
			Old age group (≥ 35 yrs.)	0.21	Middle age group × SCT non-user × folate-WT	0.4	0.31–0.51	< 0.01
		Non-user (N = 668)	Young age group (≤ 28 yrs.)	0.47				
			Middle age group (29–34 yrs.)	0.35	Middle age group × SCT non-user × folate-MUT	2.81	1.46–5.41	0.002
			Old age group (≥ 35 yrs.)	0.18				
Control (N = 870)	Folate-MUT (N = 62)	User (N = 16)	Young age group (≤ 28 yrs.)	0.5	Middle age group × SCT user × folate-WT	1.06	0.72–1.55	0.777
			Middle age group (29–34 yrs.)	0.31				
			Old age group (≥ 35 yrs.)	0.19	Middle age group × SCT user × folate-MUT	9.32	3.70–23.47	< 0.01
		Non-user (N = 46)	Young age group (≤ 28 yrs.)	0.63				
			Middle age group (29–34 yrs.)	0.26	Old age group × SCT non-user × folate-WT	1.16	0.82–1.63	0.403
			Old age group (≥ 35 yrs.)	0.11				
	Folate-WT (N = 808)	User (N = 130)	Young age group (≤ 28 yrs.)	0.48	Old age group × SCT non-user × folate-MUT	5.94	2.32–15.22	< 0.01
			Middle age group (29–34 yrs.)	0.41				
			Old age group (≥ 35 yrs.)	0.11	Old age group × SCT user × folate-WT	2.51	1.36–4.64	0.003
		Non-user (N = 678)	Young age group (≤ 28 yrs.)	0.32				
			Middle age group (29–34 yrs.)	0.58	Old age group × SCT user × folate-MUT	12.38	3.82–40.07	< 0.01
			Old age group (≥ 35 yrs.)	0.1				

Binary logistic regression analysis was performed considering maternal age, SCT use status and Folate regulator genotype as predictor variable and DS risk as outcome variable to find out the association of genotype with case and control and *P* value < 0.05 is considered statistically significant. *N* number of individuals, *OR* odd ratio, *CI* confidence interval, *Folate-WT* folate wild-type genotype, *Folate-MUT* folate polymorphic/mutant risk genotype.

### Model II: effects of SCT and genotypes in MI versus MII

This is case only analyses and we considered maternal age, maternal genotypes and maternal SCT use as predictors and meiotic errors as outcome variables. We observed more frequent DS birth among the women with folate-MUTs and SCT use in both the MI and MII groups (Table 4) in compare to any other combinations of risk factors. Moreover, the DS birth incidence was more frequent among the young age group mothers and gradually decreases with age. For example, we recorded frequency of MII error among the women who were SCT user and had folate-MUT as 0.42, 0.32 and 0.26 (Table 4) for young age group, middle age group and old age groups, respectively. Logistic regression models revealed interaction between SCT use and folate-MUTs as significant predictors of MII error (Table 4). Again, the interaction proved significant for all the age groups (OR = 21.48 for young age group and middle age group, OR = 24.1 for old age group; *P* < 0.01 for all the model). Interestingly, we found significant odds in favour of MII error for the interaction term SCT non-user × folate-MUTs in all the age groups which suggest the maternal folate-MUTs impose risk of MII NDJ even in absence of SCT.

**Table 4:** Distribution of case women stratified by meiotic errors, folate regulator genotypes, SCT use status and maternal age at conception showing interactions among various risk factors and their association with MI and MII error groups.

	Genotype	SCT use	Age	Number (%)	Regression analysis			
					Interactions (maternal age × SCT use status × polymorphism status)	OR	95% CI	*P* value
MI (N = 956)	Folate-MUT (N = 138)	User (N = 70)	Young age group (≤ 28 yrs.)	0.43	Young age group × SCT non-user × folate-WT	Reference		
			Middle age group (29–34 yrs.)	0.33				
			Old age group (≥ 35 yrs.)	0.24				
		Non-user (N = 68)	Young age group (≤ 28 yrs.)	0.41	Young age group × SCT non-user × folate-MUT	14.96	8.00–27.96	< 0.01
			Middle age group (29–34 yrs.)	0.31				
			Old age group (≥ 35 yrs.)	0.28	Young age group × SCT user × folate-WT	1.75	0.91–3.39	0.095
	Folate-WT (N = 818)	User (N = 213)	Young age group (≤ 28 yrs.)	0.46				
			Middle age group (29–34 yrs.)	0.33	Young age group × SCT user × folate-MUT	21.48	11.91–38.74	< 0.01
			Old age group (≥ 35 yrs.)	0.21	Middle age group × SCT non-user × folate-WT	1.06	0.59–1.93	0.084
		Non-user (N = 605)	Young age group (≤ 28 yrs.)	0.48				
			Middle age group (29–34 yrs.)	0.35	Middle age group × SCT non-user × folate-MUT	14.83	7.47–29.46	< 0.01
			Old age group (≥ 35 yrs.)	0.17				
MII (N = 338)	Folate-MUT (N = 237)	User (N = 144)	Young age group (≤ 28 yrs.)	0.42	Middle age group × SCT user × folate-WT	1.82	0.88–3.76	0.108
			Middle age group (29–34 yrs.)	0.32				
			Old age group (≥ 35 yrs.)	0.26	Middle age group × SCT user × folate-MUT	21.48	11.36–40.63	< 0.01
		Non-user (N = 93)	Young age group (≤ 28 yrs.)	0.42				
			Middle age group (29–34 yrs.)	0.31	Old age group × SCT non-user × folate-WT	1.77	0.93–3.39	0.083
			Old age group (≥ 35 yrs.)	0.27				
	Folate-WT (N = 101)	User (N = 36)	Young age group (≤ 28 yrs.)	0.45	Old age group × SCT non-user × folate-MUT	14.13	6.91–28.89	< 0.01
			Middle age group (29–34 yrs.)	0.33				
			Old age group (≥ 35 yrs.)	0.22	Old age group × SCT user × folate-WT	1.95	0.83–4.57	0.123
		Non-user (N = 65)	Young age group (≤ 28 yrs.)	0.42				
			Middle age group (29–34 yrs.)	0.32	Old age group × SCT user × folate-MUT	24.01	11.99–48.09	< 0.01
			Old age group (≥ 35 yrs.)	0.26				

Binary logistic regression analysis was performed considering maternal age, SCT use status and Folate regulator genotype as predictor variables and types of meiotic errors as outcome variable to find out the association of genotype with MI or MII and *P* value < 0.05 is considered statistically significant. *MI* Meiosis I, *MII* Meiosis II, *N* number of individuals, *OR* odd ratio, *CI* confidence interval, *Folate-WT* folate wild-type genotype, *Folate-MUT* folate polymorphic/mutant risk genotype.

### Model III: effects of SCT and genotype on amount of recombination in M-II NDJ group

Table 5 represents the frequency of single observed recombination events among MII women stratified by their SCT use status, folate-MUTs and age at conception. We observed reduction in double crossover frequency among the folate-MUT bearing and SCT user women than any other categories. We scored frequency of double recombinants among young age group, middle age group and old age group women as 0.13, 0.36 and 0.44, respectively for the SCT user mutant genotyped women in contrast to 0.48, 0.56 and 0.68 in the respective age categories of wild type SCT non-user group. Pair wise comparison among the tested categories was conducted using chi square tests. We found significant difference between ‘young age group folate-WT SCT non-user’ and ‘young age group folate-WT SCT user’ (*P* = 0.01), ‘young age group folate-WT SCT non-user’ and ‘young age group folate-MUT SCT non-user’ (*P* = 0.01),’ ‘young age group folate-WT SCT non-user’ and ‘young age group folate-MUT SCT user’ (*P* = 0.0006) pairs. Interestingly, maximum difference in frequency of recombinant events was recorded (*P* = 0.006) for the pairs ‘young age group folate-WT SCT non-user’ and ‘young age group folate-MUT SCT user’ with only 13% of all observed double recombination in the latter group. No other pair-wise comparisons were proved significant. Another important observation is that the differences were recorded within the young age group, not in other age groups and this suggests that the effects of ‘risk genotypes’ and ‘SCT use’ are maternal age independent.

**Table 5:** Distribution of amount of recombination events among case women stratified by folate regulator genotype, SCT use status and age at conception.

Category	SCT-use Status	Age group	N	Number of observed recombination			Chi square value and *P* value
				0	1	≥ 2	df = 2
MII mothers with folate-WT	Non-user (N = 64)	Young age group (≤ 28 yrs.)	27	N.A	0.52	0.48	**Young age group** WNU vs WU: 5.621, 0.018 WNU vs FNU: 5.617, 0.018 WNU vs FU: 11.689, 0.0006 WU vs FNU: 0.490, 0.484 WU vs FU: 0.018, 0.893 FNU vs FU: 0.765, 0.382
		Middle age group (29–34 yrs.)	20	N.A	0.44	0.56	
		Old age group (≥ 35 yrs.)	17	N.A	0.32	0.68	
	User (N = 36)	Young age group (≤ 28 yrs.)	16	N.A	0.85	0.15	
		Middle age group (29–34 yrs.)	12	N.A	0.60	0.40	**Middle age group** WNU vs WU: 0.533, 0.466 WNU vs FNU: 1.637, 0.201 WNU vs FU: 2.768, 0.096 WU vs FNU: 0.091, 0.763 WU vs FU: 0.293, 0.588 FNU vs FU: 0.091, 0.763
		Old age group (≥ 35 yrs.)	8	N.A	0.51	0.49	
MII mothers with folate-MUT	Non-user (N = 94)	Young age group (≤ 28 yrs.)	39	N.A	0.80	0.20	
		Middle age group (29–34 yrs.)	30	N.A	0.62	0.38	
		Old age group (≥ 35 yrs.)	25	N.A	0.52	0.48	**Old age group** WNU vs WU: 1.001, 0.317 WNU vs FNU: 2.108, 0.147 WNU vs FU: 2.554, 0.110 WU vs FNU: 0.010, 0.922 WU vs FU: 0.183, 0.892 FNU vs FU: 0.002, 0.961
	User (N = 144)	Young age group (≤ 28 yrs.)	58	N.A	0.87	0.13	
		Middle age group (29–34 yrs.)	48	N.A	0.66	0.36	
		Old age group (≥ 35 yrs.)	38	N.A	0.53	0.44	

Chi-square test for 3 × 2 contingency tables were performed to compare the frequency of amount of recombination events stratified by genotypes, SCT use status and maternal age within MII error group and *P* value < 0.05 was considered statistically significant. *MII* Meiosis II, *N* number of individuals, *WNU* folate-WT non-user, *WU* folate-WT user, *FNU* folate-MUT non-user, *FU* folate-MUT user, *df* degrees of freedom.

To find out true interactions among the risk factors we performed logistic regression analysis considering maternal age, maternal habits of SCT, maternal folate regulator genotypes as predictors and amount of recombination on nondisjoined Ch21 as outcome variables. In these analyses we used the interaction term ‘young age group × SCT non-user × folate-WT’ as reference. Significant interactions were recorded only in the young age group with ‘SCT use × folate-WT’ (*P* = 0.027), ‘SCT use × folate-MUT’ (*P* = 0.002) and ‘SCT non-user × folate-MUT’ (*P* = 0.021). No other models for other age groups were proved significant (Supplementary Table S6).

### Model IV: effects of SCT and genotype on spatial distribution of the observed single recombination events in MII NDJ group

Table 6 represents the distribution of single recombinant events along 21q of MII errors group stratified by maternal genotype, SCT use status and age at conception. The single observed recombination events show a change in spatial distribution pattern from the middle of the chromosome arm in the young age group to the centromere proximal position in the old age group in ‘wild type SCT non-user women’ and this observation is consistent with the findings from the previous studies^4, 5^. We observed more frequent single recombination events in the pericentromeric regions in the SCT user group as well as among the folate polymorphic genotype bearing women. This is a new finding. We scored ~ 18% of all observed single recombination events in the centromere proximal intervals 1 and 2 among the ‘young age group SCT non-user folate-WT’ women in contrast to ~ 81% of all single recombination events in ‘young age group-folate-MUT SCT user group’ (Table 6). Interestingly, the distribution pattern of single observed recombination events across the age groups remained similar among the women who were SCT user as well as had folate-MUTs. In other words, we did not observe any displacement of single recombinant events towards centromere with age among SCT user folate polymorphic genotype women. This observation is also novel. We compared the spatial distribution of single observed recombinant events among the age groups in pairwise manner through chi square test and found significant difference in the young age group between ‘folate-WT-SCT non-user’ vs ‘folate-MUT-SCT user’ (*P* < 0.0001), ‘folate-WT-SCT user’ vs ‘folate-MUT-SCT user’ (*P* < 0.0001) and ‘folate-MUT-SCT non-user’ vs ‘folate-MUT-SCTuser’ (*P* < 0.0001). Careful observation revealed both ‘SCT use’ and ‘folate-MUT’ has an effect on recombination displacement towards centromere (Table 6).

**Table 6:** Spatial distribution of single recombinant events on 21q among the case women stratified by genotype, SCT use status and maternal age at conception.

Genotype category	SCT-use Status	Maternal Age group at conception of DS	N	Long arm of nondisjoined chromosome 21						Average interval	Chi square value and *P* value
				Interval 1	Interval 2	Interval 3	Interval 4	Interval 5	Interval 6		df = 5
MII mothers with folate-WT	Non-user (N = 64)	Young age group (≤ 28 yrs.)	27	0.08	0.1	0.34	0.32	0.15	0.01	3.5	**Young age group** WNU vs WU: 2.716, 0.744 WNU vs FNU: 3.182, 0.672 WNU vs FU: 81.312. < 0.0001 WU vs FNU: 4.437, 0.488 WU vs FU: 94.159, < 0.0001 FNU vs FU: 74.385, < 0.0001
		Middle age group (29–34 yrs.)	20	0.12	0.33	0.27	0.15	0.1	0.03	3.2	
		Old age group (≥ 35 yrs.)	17	0.35	0.29	0.18	0.11	0.06	0.01	2.5	
	User (N = 36)	Young age group (≤ 28 yrs.)	16	0.03	0.11	0.35	0.33	0.16	0.02	3.52	
		Middle age group (29–34 yrs.)	12	0.1	0.35	0.24	0.17	0.09	0.05	3.21	**Middle age group** WNU vs WU: 1.095, 0.9546 WNU vs FNU: 9.512, 0.090 WNU vs FU: 42.865, < 0.0001 WU vs FNU: 9.506, 0.090 WU vs FU: 45.676, < 0.0001 FNU vs FU: 58.696, < 0.0001
		Old age group (≥ 35 yrs.)	8	0.4	0.3	0.15	0.1	0.03	0.02	2.49	
MII mothers with folate-MUT	Non-user (N = 94)	Young age group (≤ 28 yrs.)	39	0.07	0.15	0.38	0.3	0.09	0.02	3.5	
		Middle age group (29–34 yrs.)	30	0.11	0.19	0.35	0.25	0.05	0.05	3.21	
		Old age group (≥ 35 yrs.)	25	0.42	0.31	0.17	0.06	0.02	0.01	2.5	**Old age group** WNU vs WU: 9.888, 0.079 WNU vs FNU: 4.197, 0.521 WNU vs FU: 11.708, 0.039 WU vs FNU: 3.965, 0.554 WU vs FU: 2.993, 0.701 FNU vs FU: 5.043, 0.411
	User (N = 144)	Young age group (≤ 28 yrs.)	58	0.49	0.32	0.12	0.04	0.03	0.01	3.49	
		Middle age group (29–34 yrs.)	48	0.5	0.31	0.1	0.04	0.02	0.03	3.21	
		Old age group (≥ 35 yrs.)	38	0.5	0.29	0.11	0.03	0.03	0.04	1.52	

Chi-square test for 6 × 2 contingency tables were performed to compare the frequency of position of single recombination events stratified by genotypes, SCT use status and maternal age within MII error group and *P* value < 0.05 was considered statistically significant. *MII* Meiosis II, *N* number of individuals, *WNU* folate-WT non-user, *WU* folate-WT user, *FNU* folate-MUT non-user, *FU* folate-MUT user, *df* degrees of freedom.

In evaluating the effect of interactions among the predictors on the placement of single recombinant events on the we did linear regression analyses considering young age group as reference. Unlike logistic regression we considered any two predictors at a time in a given interaction model (Supplementary Table S7). We did this owing to inability of converting the position of single recombinant events into binary variables needed for data entry in logistic regression program. When considered individually, only maternal age at conception revealed as significant predictors of position of single recombinant events. Significant effects were recorded for the models ‘old age group × SCT user’ (*P* < 0.01), ‘old age group × folate-MUT’ (*P* < 0.01) and ‘SCT user × folate-MUT’ (*P* = 0.005). This observation suggests any two risk factors when present together influence effectively the position of recombination events and probably caused more centromere proximal recombinant events on 21q.

## Discussion

Complex genetic disorders usually have underpinning genetic and environmental components as causative factors. The ‘G × E model’ of human disease manifestation considers genetic makeup as ‘loaded gun’ on which ‘environmental trigger’ works. The story of chromosome NDJ is not as simple as it was thought initially. Studies have characterised the maternal age, maternal recombination anomalies on one hand and epidemiological or environmental factors on the other hand as the risk factors for NDJ. Incisive analyses have revealed complex multifactorial nature of etiology of Ch21 NDJ and the exploration of ‘G × E model’ became obvious to get a holistic view of the risk paradigm of DS birth. We for the first time ever tried to figure out the ‘G × E models’ to resolve intriguing etiology of human aneuploidy taking advantage of the largest sample cohort with DS used ever in epidemiology study and unique records regarding SCT use by Indian women. We considered all the risk factors that we analysed in our previous studies^5,11,13,27^ and these are maternal age, maternal use of SCT, maternal folate metabolic regulator genotypes.

We have tested the association of SCT use and specific folate-MUTs with the case–control and MI-MII categories stratified by maternal age at conception. The case control study demonstrated strong association of SCT use with NDJ errors among the DS bearing women with stronger effect in the young age group (*P* < 0.0001), than the middle age group women (*P* = 0.02) and no association in old age group women (Supplementary Table S2). This observation supports the notion that SCT challenges faithful chromosome segregation irrespective of age at conception of the women.

We analysed maternal genotypes of selective mutant variants of the folate metabolic regulator genes, namely MTR A2756G, MTRR A66G, and MTHFR C677T & MTHFR A1298C. These variants were reported to be associated with DS birth in different ethnic populations^28–30^. We tested association of all four variants and obtained significant elevated odds in favour of the MII error group over the MI error group for homozygous minor and heterozygous genotypes of all four polymorphic sites except the ‘GG’ of MTR A2756G (Table 2).

We designed several ‘G × E models’ and conducted logistic regression analyses considering maternal age, maternal folate regulator genotypes, maternal habits of SCT use and interaction terms among them as predictors for different outcome variables like incidence of DS birth, types of meiotic errors, recombination amount and position of single recombinant events on 21q. In case–control analyses we obtained significant effects for the interaction terms ‘young age group × SCT user × folate-MUT’, ‘middle age group × SCT user × folate-MUT’ and ‘old age group × SCT user × folate-MUT’ (Table 3) on the incidence of DS birth. This result supports the hypothesis that the interactions between SCT use and folate-MUT increases risk of DS birth incidence in maternal age independent manner. The rationale of this inference is that any risk factor that is age dependent shows highest frequency in young age group which is not otherwise suffers from age related challenges. On contrary, age related risk factors show increasing frequency with age.

In the second model which is a case only study we analysed the effects of all the risk factors and their interaction terms on the types of meiotic error, i.e., MI and MII. As the biology of MI and MII is different we performed this analysis to get insight into the imperilment incurred by risk factors on the specific mechanism of meiotic chromosome segregation. We observed highest frequency of the folate-MUTs among the young age group women within the MII group with gradual decrease in frequency with age (Table 4). This observation confirms our previous^27^ hypothesis that folate metabolic regulator polymorphisms are the risk factors for MII errors, probably affect chromatid separation at advanced phase of oogenesis and the effects is probably maternal age independent following the rationale explained above. In regression analyses for various interactions among the predictors we observed many folds elevated odds in favour of MII errors (Table 4). This observation suggests combined effect of maternal folate-MUTs and maternal exposure to SCT elevates risk of MII NDJ in maternal age independent manner. Interestingly, we obtained significant interaction between ‘folate-MUT’ and ‘SCT non-user’ group for all the age categories, though the odds were less than the odds estimated for the interaction term ‘SCT use × folate-MUT’. This observation in turn suggests that the folate-MUTs are significant risk factors for MII errors for all age groups and the risk is exacerbated in association with maternal SCT use. This hypothesis needs further evidence in favour to be confirmed.

In analysing effects of SCT use and folate-MUT on the amount of recombination we observed gradual decrease of single recombinant events and gradual increase in frequency double recombinant events with advancing age of MII women when stratified with age at conception and SCT use status. This observation is novel (Table 5) and the results suggest SCT use and folate-MUT probably reduce the frequency of recombinant events on 21q and the effect is maternal age independent. That is why significant difference is obtained only in young age group. This hypothesis is further supported by the results of logistic regression models considering above mentioned genetic, epidemiological factors and their interaction terms as predictors and amount of recombinant as outcome (Supplementary Table S6). This result intuitively suggests both the ‘folate-MUT’ and ‘SCT-use’ reduce the recombinant event on 21q among the young age group women independently and their interaction has stronger effects which imperil sister chromatid separation at MII. Further, it can be inferred that SCT-use and folate-MUT affect young age group more than old age group.

Lastly, we analysed the effects of ‘SCT use’ and ‘folate polymorphic genotype’ on the spatial distribution of single recombinant events on 21q of MII events. We observed almost similar distribution of single recombinant events in the pericentromeric interval 1 across the age groups in ‘folate-MUT-SCT use category’ (Table 6). This is a novel observation. This suggests ‘SCT use’ and ‘folate-MUT’ have combined effects in displacement of recombinant events towards centromere of 21q in all age groups (Supplementary Table S7). This outcome suggests any two of the three risk factors i.e., ‘SCT use’, ‘folate-MUT’ and ‘old age group’ when present together increase risk of incorrectly positioned single recombinant events towards centromere which probably cause chromosome entanglement and non-separation of sister chromatids in MII. Though, this hypothesis is speculative and need further incisive study to be confirmed. It is very difficult to justify why distribution of single recombinant events in the interval 1 is almost similar across the age group in one hand, but significant interaction was obtained with the old age group only in the regression model on the other hand. It may be possible that age of women has stronger effects on the anomalous positing of single recombinant events on 21q than other age groups.

In summary, we for the first time ever demonstrated interactions between maternal ‘SCT use’ and ‘maternal folate-MUT’that increase risk of reduced recombination and pericentromeric single recombinant events on the Ch21, which nondisjoined at MII. This effect is probably maternal age independent. Previously we^13^ observed maternal ‘SCT use’ was associated with the MII errors and reduced the recombination frequency. Therefore, the outcome of the present study confirms the notion that SCT in interaction with maternal folate polymorphic genotypes increases the chance of anomalous placement of recombination. This observation is novel as we did not find this association in any other published literatures. More incisive analyses are needed to resolve this issue.

Limitation of our present study is to overlook the other polymorphisms (besides the tested four) of the folate regulator genes in maternal genome which may have some confounding effects in the present outcome. But again, it is difficult at moment to differentiate the contribution of the tested four polymorphisms on observed incidence of meiotic errors from the share of the other genetic risk factors that we did not in the study. Moreover our study suffers from lack of supportive evidence from other studies. Nevertheless, our work is the pioneer attempt to address the critical issue of interaction between genetic and environmental risk factors in the intriguing etiology of Ch21 NDJ and birth of child with DS. This result will provide foundation to design ‘G × E models’ for other human aneuploidy and complex genetic disorders. These findings bring us a significant step closer towards understanding the cause Ch21NDJ in the human oocyte.

## Methods

### Ethics declarations

The experimental protocols and analyses were reviewed and approved by the ethics committee constituted by the University of Calcutta and IPGMER, Kolkata, India. The study was conducted following ethical compliance as outlined by declaration of Helsinki and Indian Council of Medical Research (ICMR).

### Consent statement

Informed consents in the pre-printed questionnaire were taken from all participating families.

### Sample collections and epidemiological data recordings

#### Trisomic samples

Families were referred to the laboratory from Kolkata and its surrounding Medical Colleges and Hospitals by the clinician collaborators. A total of 1294 families, each with single child with DS having free trisomy 21 were included in this study. Eligibility criteria for selecting Trisomic samples were availability of complete set of tissue samples from DS family trios, free trisomy 21 and live born child with DS as determined by classical karyotyping at our laboratory and complete information regarding maternal life style, especially periconceptional Smokeless Chewing Tobacco (SCT) use and related issues. Interviews of mothers were taken very privately, in person, after obtaining full consents. A pre-printed, extensive set of questions was used for each family to collect detailed family history, information about lifestyle, history of miscarriage or abnormal pregnancy outcome if any and other relevant epidemiological details. The confidentiality of all information was maintained very carefully at our laboratory. The participating cohort sample consisted chiefly of Bengali-speaking families from West Bengal; the majority believed in either Hinduisms or Islam.

#### Controls

A total of 870 families, each having healthy euploid (2n = 46, XX or 46, XY) infant were recruited as the controls and their karyotype was confirmed through classical karyotyping at our laboratory. The controls were identified and selected randomly from healthy new-borns without any birth defect from the enrolled patient databases and birth registers of the hospitals that provided the cases to ensure maximum similarity in demographic attributes between the participating cases and controls. The minimum eligibility criteria for enrolment of control family were the completion of the maternal questionnaire and availability of at least maternal and child tissue samples.

#### Tissue collection

Tissue samples were donated voluntarily by the families following their understanding of use of tissue in academic research and its importance as explained by the clinician or by the first author of this manuscript. Informed consent for tissue uses in research was obtained from each of the participating families. Tissue samples were collected from trisomic family trios (Cases) i.e., DS child, father and mother as well as from the families with euploid baby (Controls). Nearly 2 ml of venous blood samples were collected by venipunctute method in EDTA coated vacutainer tubes and were stored in − 20 °C refrigerator till DNA isolation was done. The highest biomedical ethics were maintained during the study.

### Genotyping and recombination scoring

#### DNA isolation

Genomic DNA was isolated from whole blood samples by using QIAamp Blood Mini Kit (QIAGEN, Hilden, Germany) according to the manufacturer's instructions.

#### Detection of parental origin and stage of meiotic nondisjunction

**Figure 1:**
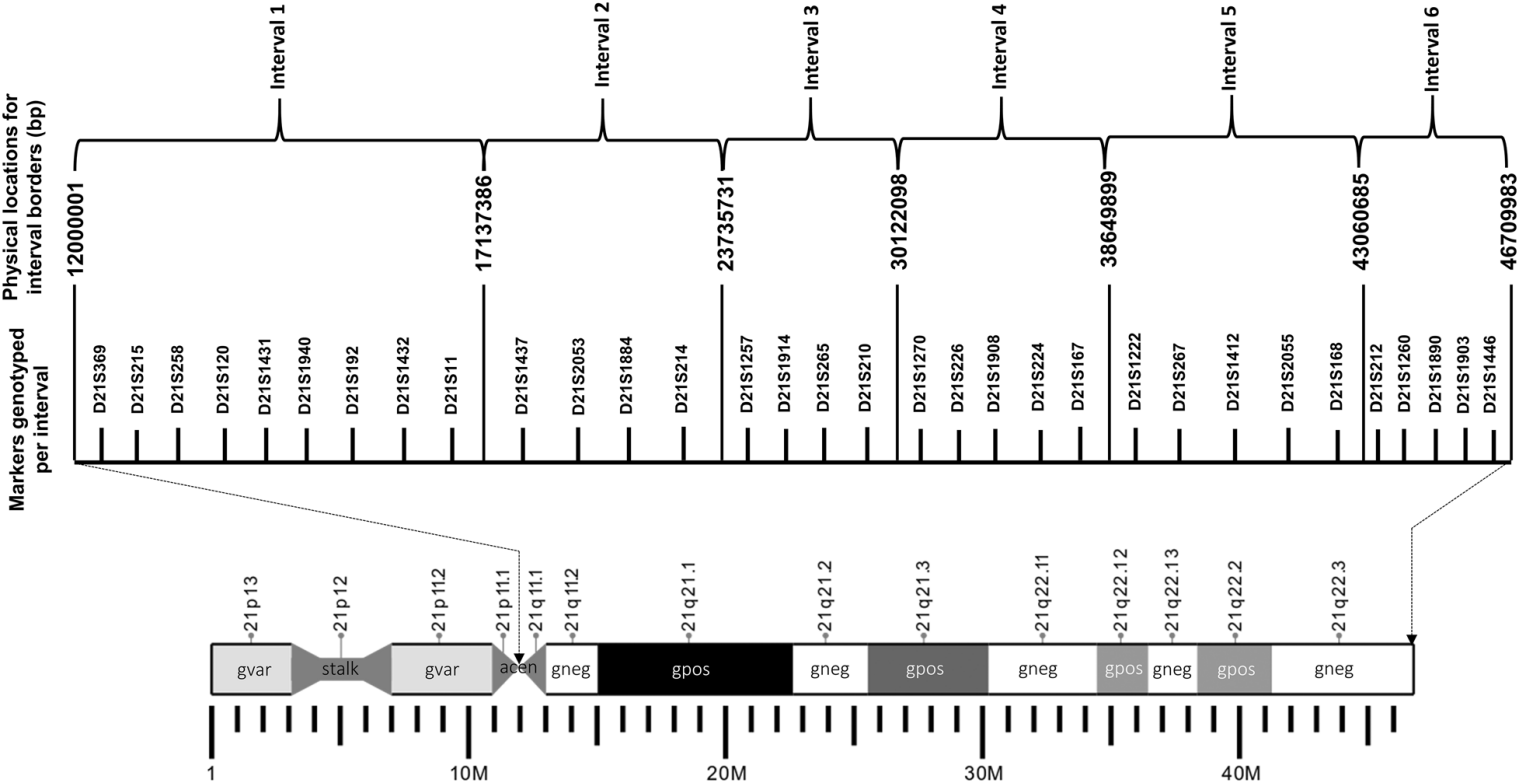
Universal STR markers used to define the origin of the meiotic error and determine the recombination profile. Panel of 32 universal STR markers were used to determine the parental origin (Maternal or Paternal) of error as well as type of meiotic error (Meiosis I or Meiosis II). Only cases in which the error was maternal in origin were included in this study. Once the origin of the error was defined, this genotyping information was used to determine the number and location of recombination (i.e., recombination profile). 21q was divided into six intervals of approximately equal physical length. Each observed recombinant was defined as being located in one of six defined intervals.

Each participating family was genotyped with a panel of Ch21q specific short tandem repeat (STR) markers, covering from the pericentromeric region to the telomere (Fig. 1). These markers were used for determining parental origin of error as well as meiotic stage of error of NDJ. The parental origin of NDJ was determined by detecting the contribution of parental alleles to the probands for multiple markers. We inferred a MI error when the parental heterozygosity of the pericentromeric markers was retained in the trisomic child (i.e., the marker was ‘‘nonreduced”) and MII error when parental heterozygosity was ‘‘reduced’’ to homozygosity. MII events with no evidence of recombination were considered to be mitotic errors and were excluded, as described elsewhere^4^. Earlier study has reported that some proportion of so-called MII errors actually originate at meiosis I. Despite this fact, we took the conventional approach to define “MI” and ‘‘MII’’ errors as we did in our previous studies^5,13^. The determination of MI or MII was done blinded without knowing status of the epidemiological risk exposure of the women and their genotypes of selected polymorphic loci of folate metabolic regulators.

#### Characterization of observed recombinant events on 21q

Standard methods for trisomic data^31^ were used for scoring recombination events. This recombination scoring is possible even though only one child is genotyped, owing to the fact that trisomic child bears two copies of Ch21 and thus is essentially a self-contained sibling pair for that chromosome. We did not estimate recombination in the control subjects as conventional recombination scoring requires either grandparents or at least two siblings which were unavailable for all the subjects. Thus, recombination was considered only in the case-only phase of our analysis.

Apart from four pericentromeric markers mentioned earlier, all additional markers were used to divide entire chromosome 21q into six intervals to monitor the recombinant events. After genotyping we recorded the marker status as reduced (R), nonreduced (N) or uninformative (U) and arranged them successively in linear direction from centromere to telomere of 21q arm. The recombination event was scored whenever we observed a transition of two successive markers from nonreduced (N) to reduced (R) or reduced (R) to nonreduced (N) in the ordered panel of makers along the 21q.

#### Detection of Folate metabolic regulator polymorphisms

Bi-directional Sanger sequencing method was used to determine the maternal genotype for four polymorphic sites namely, MTR A2756G (rs1805087), MTRR A66G (rs1801394), MTHFR C677T (rs1801133) and MTHFR A1298C (rs1801131). All primers were designed by Primer3 (v.0.4.0) program and tested by OligoAnalyzer tool from Integrated DNA Technology (IDT). The PCR amplification was performed in a 30 μl reaction volume containing 50–100 ng of DNA, 1 μl of each primer (10 mmol/L), 0.2 μl of deoxyribonucleotide triphosphate mix (dNTPs, 10 mmol/L; Invitrogen Carlsbad, CA, USA), 1.5 μl magnesium chloride (MgCl2, 50 mmol/L), 1 × PCR reaction buffer and 0.8 μl of Taq Polymerase (5 units/1 μl; Invitrogen, California, USA). Sanger sequencing was done using a Taq Dye Deoxy Terminator sequencing kit (Applied Biosystems, Foster City, USA) with an ABI Prism 377 DNA sequencer (Applied Biosystems, Foster City, USA). The primer sets used for Sanger sequencing are:

- MTR A2756G (rs1805087): FORWARD-5′ GTCTCCCAGAAACCAGTCAA 3′/REVERSE 5′ TCTAGCACAGCCCCTAACAC 3′,
- MTRR A66G (rs1801394): FORWARD-5′ TCGTACACTCTCCTTAATTTGATG 3′/REVERSE-5′ GATTCAAGAGGTGGAAAGCA 3′,
- MTHFR C677T (rs1801133): FORWARD-5′ ACAGTGTGGGAGTTTGGAG 3′/REVERSE 5′ AGTTCTGGACCTGAGAGGAG 3′,
- MTHFR A1298C (rs1801131): FORWARD-5′ CCTCCAGACCAAAGAGTTAC 3′/REVERSE-5′ CTGTGAGTTGATGGTGAGG 3′,

The reaction condition for sequencing PCR was: initial denaturation at 94 °C (5 min), annealing at 55 °C (30 s), elongation at 72 °C (30 s) and a final elongation at 72 °C (5 min) for a total of 40 cycles.

### Statistical analysis

We employed Fisher’s exact test to determine differences in allele and genotype distributions between case and control groups and to verify that allele frequencies were in Hardy–Weinberg equilibrium. Odds ratios with respective 95% Confidence Interval (CI) were calculated for association study. Chi square (χ^2^) test was performed to compare MI: MII ratios. Chi square (χ^2^) tests were used to compare other epidemiological parameters between MI and MII case and control groups and t-tests were used to compare the differences between mean maternal and parental age at conception, preconceptionmaternal folic acid intake amount. Binary logistic regression analysis were performed to study a variety of questions regarding interaction among maternal age group, SCT use status and folate polymorphic genotype with DS risk, maternal meiotic errors and amount of recombination. Linear regression analysis was performed to study interactions among maternal age group, SCT use status and folate polymorphic genotype with position of single recombination events. All the analyses were done using software package STATA 13 (StataCorp LP, College Station, Texas). We have described the folate regulator gene mutations or polymorphisms as ‘folate polymorphic genotype’ for simplicity in rest of the manuscript.

## Supplementary Information

Supplementary Tables.

## Data Availability

All data generated or analysed during this study are included in this article (and its Supplementary Information files).
