# Supplementary material for: Understanding etiology of chromosome 21 nondisjunction from gene × environment models
Source: Sci Rep. 2021 Nov 17;11:22390. doi: 10.1038/s41598-021-01672-x (PMC8599692; doi:10.1038/s41598-021-01672-x)
Supplement: Supplementary file 1 — Supplementary Tables. [file 41598_2021_1672_MOESM1_ESM.pdf]

Supplementary Table S1

Supplementary Table S1. Effect of SCT use among Case & Control group and their association with DS birth risk. Fisher's exact test for 2 × 2 contingency tables were performed and P value <0.05 was considered statistically significant. N: number of individuals; CI: Confidence Interval.

SCT use status	Control (N=870)	Case (N=1294)	ODD ratio	95% CI	P value
**Non-user**	0.83	0.64	2.772	2.245 - 3.424	<0.0001
**User**	0.17	0.36			

Supplementary Table S2

Supplementary Table S2. Association of SCT use status with cases and controls startified by maternal age group.
Fisher's exact test for 2 × 2 contingency tables were performed and P value <0.05 was considered statistically significant. N: number of individuals; OR: Odd Ratio; CI: Confidence Interval.

Characteristic		Young age group (≤28 Years)			Middle age group (29-34 Years)			Old age group (≥35 Years)		
All Maternal Case and Control (N=2164)		Case (N=589)	Control (N=410)	OR (95%CI), P value	Case (N=438)	Control (N=293)	OR (95%CI), P value	Case (N=267)	Control (N=167)	OR (95%CI), P value
SCT use status	Non-user	0.66	0.79	1	0.65	0.73	1	0.6	0.63	1
	User	0.34	0.21	1.996 (1.468-2.643), <0.0001	0.35	0.27	1.458 (1.056-2.015), 0.024	0.4	0.37	1.133 (0.760-1.686), 0.608

Supplementary Table S3

Supplementary Table S3. Allele and Genotype frequencies of MTR: rs1805087, MTRR: rs1801394, MTHFR: rs1801133 and rs1801131 and association with DS birth risk. Fisher's exact test for 2 × 2 contingency tables were performed and P value <0.0125 was considered statistically significant after Bonferroni Correction test. N: number of individuals; MII: Meiosis II; NDJ: Nondisjunction; CI: Confidence Interval.

SNPs	Allele	Allele frequency		OR (95% CI)	P- value	Genotype	Control (N = 870)	MII NDJ (N = 338)	Odd ratio (95% CI)	P- value
		Control (N = 870)	MII NDJ (N = 338)							
MTR A2756G (rs1805087) A > G	A	0.89	0.78	2.293 (1.811 - 2.903)	< 0.0001	AA	0.8	0.57	Reference	
	G	0.11	0.22			AG	0.17	0.41	3.387 (2.557 - 4.487)	<0.0001
						GG	0.03	0.02	0.5548 (0.1913 - 1.609)	0.3669
						AG OR GG	0.2	0.43	2.964 (2.256 - 3.893)	<0.0001
						AA vs AG+GG			2.964 (2.256 - 3.893)	<0.0001
						AA+AG vs GG			0.3911 (0.1354 - 1.130)	0.0969
MTRR A66G (rs1801394) A > G	A	0.88	0.76	2.282 (1.816 - 2.867)	< 0.0001	AA	0.78	0.53	Reference	
	G	0.12	0.24			AG	0.2	0.45	3.314 (2.522 - 4.354)	< 0.0001
						GG	0.02	0.02	1.562 (0.6378 - 3.825)	0.3153
						AG OR GG	0.22	0.47	3.158 (2.417 - 4.125)	< 0.0001
						AA vs AG+GG			3.158 (2.417 - 4.125)	< 0.0001
						AA+AG vs GG			1.061 (0.4360 - 2.583)	1
MTHFR C677T (rs1801133) C >T	C	0.9	0.88	1.225 (0.926 - 1.621)	0.1771	CC	0.85	0.81	Reference	
	T	0.01	0.12			CT	0.11	0.13	1.236 (0.8429 - 1.813)	0.3129
						TT	0.04	0.06	1.541 (0.8744 - 2.716)	0.1618
						CT OR TT	0.15	0.19	1.318 (0.9477 - 1.832)	0.1166
						CC vs CT+TT			1.318 (0.9477 - 1.832)	0.1166
						CC+CT vs TT			1.500 (0.8532 - 2.639)	0.1672
MTHFR A1298C (rs1801131) A >C	A	0.78	0.64	1.988 (1.638 - 2.414)	< 0.0001	AA	0.58	0.35	Reference	
	C	0.22	0.36			AC	0.39	0.59	2.525 (1.935 - 3.294)	< 0.0001
						CC	0.03	0.06	3.292 (1.777 - 6.099)	0.0002
						AC OR CC	0.42	0.65	2.580 (1.987 - 3.349)	< 0.0001
						AA vs AC+CC			2.580 (1.987 - 3.349)	< 0.0001
						AA+AC vs CC			2.042 (1.124 - 3.710)	0.0279

Supplementary Table S4

Supplementary Table S4. Co-occurrence of maternal polymorphic variants and associated risk for Down syndrome birth. Fisher's exact test for 2 × 2 contingency tables were performed and P value <0.006 was considered statistically significant after Bonferroni correction test. N: number of individuals; OR: Odd Ratio; CI: Confidence Interval.

**MTR A2756G**	**MTRR A66G**	**Control (N = 870)**	**MII NDJ (N = 338)**	**OR**	**95% CI**	**P value**
AA	AA	0.59	0.36	Reference		
AA	AG	0.26	0.21	1.3	0.93 - 1.81	0.141
AA	GG	0.002	0.006	4.139	0.58 - 29.69	0.175
AG	AA	0.19	0.17	1.43	1.00 - 2.05	0.056
AG	AG	0.01	0.21	3.378	2.332- 4.894	<0.0001
AG	GG	0.018	0.028	2.328	1.01 - 5.40	0.069
GG	AA	0.01	0.009	1.38	0.37 - 5.18	0.711
GG	AG	—	—	—	—	—
GG	GG	—	—	—	—	—
**MTR A2756G**	**MTHFR C677T**	**Control (N = 870)**	**MII NDJ (N = 338)**	**OR**	**95% CI**	**P value**
AA	CC	0.68	0.48	Reference		
AA	CT	0.099	0.091	1.273	0.81 – 2.00	0.336
AA	TT	0.019	0.03	2.146	0.96 – 4.78	0.062
AG	CC	0.192	0.348	2.578	1.92- 3.46	< 0.0001
AG	CT	0.038	0.047	1.769	0.95 – 3.30	0.077
AG	TT	0.002	0.009	5.472	0.91 – 33.04	0.072
GG	CC	0.008	0.015	2.606	0.82 – 8.32	0.149
GG	CT	—	—	—	—	—
GG	TT	—	—	—	—	—
**MTR A2756G**	**MTHFR A1298C**	**Control (N = 870)**	**MII NDJ (N = 338)**	**OR**	**95% CI**	**P value**
AA	AA	0.42	0.3	Reference		
AA	AC	0.351	0.259	1.043	0.75 – 1.44	0.805
AA	CC	0.042	0.056	1.907	1.05 – 3.47	0.041
AG	AA	0.125	0.121	1.359	0.89 - 2.07	0.181
AG	AC	0.072	0.269	5.22	3.54 – 7.71	<0.0001
AG	CC	0.008	0.016	2.581	0.80 – 8.31	0.15
GG	AA	0.015	—	0.133	0.01 – 2.26.	0.08
GG	AC	0.001	0.007	7.228	0.65 - 80.57	0.123
GG	CC	—	—	—	—	—
**MTRR A66G**	**MTHFR C677T**	**Control (N = 870)**	**MII NDJ (N = 338)**	**OR**	**95% CI**	**P value**
AA	CC	0.62	0.46	Reference		
AA	CT	0.075	0.09	1.605	1.00 – 2.56	0.053
AA	TT	0.019	0.125	8.591	4.76 – 15.52	<0.0001
AG	CC	0.195	0.393	2.721	2.04 - 3.63	< 0.0001
AG	CT	0.037	0.033	1.195	0.59 - 2.43	0.578
AG	TT	0.007	0.009	1.739	0.43 – 7.04	0.428
GG	CC	0.009	0.022	3.043	1.09 – 8.53	0.054
GG	CT	—	—	—	—	—

GG	TT	–	–	–	–	–
**MTRR A66G**	**MTHFR A1298C**	**Control (N = 870)**	**MII NDJ (N = 338)**	**OR**	**95% CI**	**P value**
AA	AA	0.73	0.31	Reference		
AA	AC	0.337	0.264	1.837	1.34 – 2.52	0.0002
AA	CC	0.041	0.032	1.848	0.91 – 3.75	0.091
AG	AA	0.129	0.136	2.484	1.66 - 3.71	< 0.0001
AG	AC	0.083	0.223	6.3	4.29 – 9.25	<0.0001
AG	CC	0.037	0.05	3.213	1.72– 5.99	0.0007
GG	AA	0.013	0.02	3.848	1.46 - 10.15	0.01
GG	AC	0.01	0.012	2.688	0.81 - 8.89	0.105
GG	CC	–	–	–	–	–
**MTHFR C677T**	**MTHFR A1298C**	**Control (N = 870)**	**MII NDJ (N = 338)**	**OR**	**95% CI**	**P value**
CC	AA	0.47	0.392	Reference		
CC	AC	0.381	0.31	0.983	0.73 – 1.32	0.94
CC	CC	0.062	0.072	1.377	0.82 – 2.32	0.264
CT	AA	0.05	0.039	0.912	0.48 - 1.75	0.872
CT	AC	0.043	0.065	1.842	1.05 – 3.24	0.041
CT	CC	0.009	0.015	1.94	0.62 – 6.02	0.325
TT	AA	0.019	0.024	1.46	0.62 – 3.46	0.476
TT	AC	0.009	0.009	1.16	0.30 - 4.45	0.735
TT	CC	–	–	–	–	–

Supplementary Table S5

Supplementary Table S5. Risk of Down syndrome birth among women increases with the synergistic effects of increasing number of minor allelels in all four tested variants of folate regulators. Fisher's exact test for 2 × 2 contingency tables were performed and P value <0.003 was considered statistically significant after Bonferroni Correction test. N: number of individuals; OR: Odd Ratio; CI: Confidence Interval.

Maternal genotype combinations	Risk Allele	Control (N = 870)	MII NDJ (N = 338)	OR	95% CI	P value
MTR 2756 AA vs MTRR 66 AA vs MTHFR 677 CC vs MTHFR 1298 AA	0	193	40	Reference		
MTR 2756 AA vs MTR 66 AA vs MTHFR 677 CC vs MTHFR 1298 AA or AC	1	105	23	1.057	0.60 - 1.86	0.885
MTR 2756 AA vs MTRR 66 AA vs MTHFR 677 CC vs MTHFR 1298 AA or CC	2	85	27	1.533	0.88 - 2.66	0.146
MTR 2756 AA vs MTRR 66 AA vs MTHFR 677 CC vs MTHFR 1298 AC or CC	3	149	53	1.716	1.08 - 2.73	0.026
MTR 2756 AA vs MTRR 66 AA vs MTHFR 677 CC or CT or MTHFR 1298 AC or CC	4	111	49	2.13	1.32 - 3.44	0.002
MTR 2756 AA vs MTR 66 AA vs MTHFR 677 CC or TT or MTHFR 1298 AC or CC	5	84	47	2.7	1.65 - 4.42	0.0001
MTR 2756 AA vs MTRR 66 AA vs MTHFR 677 CT or TT or MTHFR 1298 AC or CC	6	56	41	3.53	2. 08 - 5.99	<0.0001
MTR 2756 AA vs MTRR 66 AA or AG or MTHFR 677 CT or TT or MTHFR 1298 AC or CC	7	38	38	4.825	2.75 - 8.48	<0.0001
MTR 2756 AA vs MTRR 66 AA or GG or MTHFR 677 CT or TT or MTHFR 1298 AC or CC	8	21	32	7.352	3.85 - 14.05	<0.0001
MTR 2756 AA vs MTRR 66 AG or GG or MTHFR 677 CT or TT or MTHFR 1298 AC or CC	9	16	29	8.745	4.35 - 17.60	<0.0001
MTR 2756 AA or AG vs MTRR 66 AG or GG or MTHFR 677 CT or TT or MTHFR 1298 AC or CC	10	14	27	9.305	4.48 - 19.31	<0.0001
MTR 2756 AA or GG vs MTRR 66 AG or GG or MTHFR 677 CT or TT or MTHFR 1298 AC or CC	11	9	22	11.794	5.06 - 27.52	<0.0001
MTR 2756 AG or GG vs MTRR 66 AG or GG or MTHFR 677 CT or TT or MTHFR 1298 AC or CC	12	4	14	16.89	5.28 - 54.00	<0.0001

Supplementary Table S6

Supplementary Table S6. Interaction among various risk factors and their association with amount of recombination events. Binary logistic regression analysis was performed considering maternal age, SCT use status and folate regulator genotype as predictor variables and amount of recombination as outcome variable and P value <0.05 was considered statistically significant. OR: Odd Ratio; CI: Confidence Interval; folate-WT: Wild-type genotype; folate-MUT : Folate polymorphic / mutant risk genotype.

Interactions (maternal age × SCT use status × polymorphism status)	OR	95% CI	P value
Young age group × SCT non-user × folate-WT	Reference		
Young age group × SCT non-user × folate-MUT	0.28	0.09 – 0.82	0.021
Young age group × SCT user × folate-WT	0.15	0.30 – 0.81	0.027
Young age group × SCT user × folate-MUT	0.19	0.07 – 0.54	0.002
Middle age group × SCT non-user × folate-WT	1.44	0.46 – 4.52	0.537
Middle age group × SCT non-user × folate-MUT	0.66	0.23 – 1.91	0.441
Middle age group × SCT user × folate-WT	0.77	0.19 – 3.04	0.708
Middle age group × SCT user × folate-MUT	0.52	0.20 – 1.38	0.19
Old age group × SCT non-user × folate-WT	2.58	0.71 – 9.37	0.148
Old age group × SCT non-user × folate-MUT	0.99	0.33 – 2.95	0.991
Old age group × SCT user × folate-WT	1.08	0.22 – 5.21	0.93
Old age group × SCT user × folate-MUT	0.97	0.36 – 2.60	0.951

Supplementary Table S7

Supplementary Table S7. Interaction among various risk factors and their association with position of single recombinant events. Linear regression analysis was performed considering maternal age, SCT use status and folate regulator genotype as predictor variables and position of single recombination events as outcome variable and P value <0.05 was considered statistically significant. Coef.: Regression coefficients; CI: Confidence Interval; folate-WT: Wild-type genotype; folate-MUT : Folate polymorphic / mutant risk genotype.

Interactions (maternal age × SCT use status × polymorphism status)	Coef.	95% CI	P value
**Young age group**	Reference		
**Middle age group**	-0.3052891	-.3581 – -.2525	<0.01
**Old age group**	-0.7797251	-.8364 – -.7231	<0.01
**SCT non-user**	Reference		
**SCT user**	-0.001052	-.0373 – .0352	0.955
**folate-WT**	Reference		
**folate-MUT**	-0.0132563	-.0526 – .0261	0.508
**Young age group × SCT non-user**	Reference		
**Middle age group × SCT user**	0.0040557	-.0513 – .0594	0.885
**Old age group × SCT user**	-0.7217861	-.7807 – -.6628	<0.01
**Young age group × folate-WT**	Reference		
**Middle age group × folate-MUT**	0.0188699	-.0411 – .0788	0.536
**Old age group × folate-MUT**	-0.3699654	-.4348 – -.3052	<0.01
**SCT non-user × folate-WT**	Reference		
**SCT non-user × folate-MUT**	-0.0007858	-.1986 – .1971	0.994
**SCT user × folate-WT**	0.0462393	-.2080 – .3005	0.721
**SCT user × folate-MUT**	-0.260844	-.4437 – .0780	0.005
